# An experimental examination of cognitive processes and response inhibition in patients seeking treatment for buying-shopping disorder

**DOI:** 10.1371/journal.pone.0212415

**Published:** 2019-03-06

**Authors:** Birte Vogel, Patrick Trotzke, Sabine Steins-Loeber, Giulia Schäfer, Jana Stenger, Martina de Zwaan, Matthias Brand, Astrid Müller

**Affiliations:** 1 Department of Psychosomatic Medicine and Psychotherapy, Hannover Medical School, Hannover, Germany; 2 Department of General Psychology: Cognition, Center for Behavioral Addiction Research (CeBAR), University Duisburg-Essen, Duisburg, Germany; 3 Department of Clinical Psychology, University Bamberg, Bamberg, Germany; 4 Department of Psychiatry, University of Basel, Basel, Switzerland; Ariel University, ISRAEL

## Abstract

There is an ongoing debate about whether buying-shopping disorder (BSD) should be acknowledged as a behavioral addiction. The current study investigated if mechanisms that play a prominent role in disorders due to substance use or addictive behaviors are relevant in BSD, particularly cue reactivity, craving, cognitive bias and reduced inhibitory control regarding addiction-relevant cues. The study included 39 treatment-seeking patients with BSD and 39 healthy control (HC) participants (29 women and 10 men in each group). Subjective responses toward buying/shopping-relevant visual cues were compared in patients vs. control participants. Experimental paradigms with neutral and semi-individualized buying/shopping-related pictures were administered to assess attentional bias, implicit associations and response inhibition with respect to different visual cues: Dot-probe paradigm (DPP), Implicit Association Task (IAT), Go/nogo-task (GNG). The severity of BSD, craving for buying/shopping, and symptoms of comorbid mental disorders (anxiety, depressive and hoarding disorders) were measured using standardized questionnaires. The BSD-group showed more general craving for buying/shopping, stronger subjective craving reactions towards buying/shopping-related visual cues, and more symptoms of anxiety, depression and hoarding disorder than control participants. Task performance in the DPP, IAT and GNG paradigm did not differ between the two groups. The present findings confirm previous research concerning the crucial role of craving in BSD. The assumption that attentional bias, implicit associations and deficient inhibitory control with respect to buying/shopping-related cues are relevant in BSD could not be proven. Future research should address methodological shortcomings and investigate the impact of acute psychosocial stress and present mood on craving responses, cognitive processing, and response inhibition in patients with BSD.

## Introduction

Buying-shopping disorder (BSD) is characterized by extreme preoccupation with buying/shopping, an overwhelming urge to possess consumer goods, recurrent purchases of unnecessary things and irrational beliefs about material possessions [1–4]. According to patients’ reports, the excessive buying/shopping episodes generate a short-term reward (i.e. pleasure, fun, thrill, excitement, etc.). With the progression of BSD, these episodes become habitual and serve to manage negative feelings (e.g. anxiety, depression, tension, frustration, boredom) and to escape distress [5,6]. Although the harmful spending behavior results in adverse consequences (e.g. debts, familial discord, clutter due to hoarded consumer items, comorbid mental disorders), repeated efforts to cut down buying/shopping activities remain unsuccessful [2]. In some cases, violations of the rights of others (e.g. deception, embezzlement) may occur in order to continue overspending despite indebtedness.

Population-based surveys of BSD have been carried out since more than 30 years [7,8]. They provided evidence that BSD is a public health problem across different cultures [9–13]. Results of a meta-analysis revealed a propensity towards BSD of about 5% in representative adult samples [14], which indicates the clinical need of any advance in basic research. BSD is associated with psychiatric comorbidity, including anxiety, depressive and hoarding disorder [1,3,15,16]. The release version of the 11^th^ edition of the International Classification of Diseases (ICD-11) [17] does not include BSD as an independent mental health condition, but lists”compulsive buying-shopping disorder” as an example in the residual category”Other specified impulse control disorders” (category 6C7Y). Impulse control disorders “…should be defined by the repeated failure to resist an impulse, drive, or urge to perform an act that is rewarding to the person (at least in the short-term), despite longer term harm either to the individual or others” according to the ICD-11 working group on obsessive-compulsive disorder and related disorders [18]. Phenomenologically, BSD seems to meet these impulse control disorder criteria [1–3]. However, recent research findings suggest that BSD should rather be considered a candidate for the proposed ICD-11 category”Other specified disorders due to addictive behaviors” [19,20]. Analogous to substance use disorders and gambling disorder, experimental studies emphasized the prominent role of cue-induced craving and reward processing, attentional bias, dysfunctional decision-making and deficits in response inhibition in BSD [21–30].

Cue-reactivity and craving are acknowledged as underlying mechanisms in the development and maintenance of substance use disorders [31] and behavioral addictions [28]. According to the incentive sensitization theory of addiction, the frequent presentation of substance-related stimuli evokes an attentional bias and implicit (automatic) positive associations towards these stimuli due to classical conditioning, which results in cue-induced craving [32]. Similar to the repeated administration of a certain substance, recurrent activity in a rewarding behavior may strengthen the motivational properties, leading to subjective craving for this behavior. As in substance use disorders, cue-induced craving for certain behaviors is supposed to be interrelated with an attentional bias and positive implicit cognitions towards behavior-related cues [33–37]. With respect to BSD, it can be assumed that due to the immediate experience of gratification while buying/shopping, specific cues (e.g. shopping malls/websites, brands, commercials, price promotions) may become related to the positive reinforcing features of buying/shopping (“liking”), making these cues attractive. Subsequently, the confrontation with these cues may elicit strong craving for buying/shopping (“wanting”) [38] that is associated with positive cognitive responses and appetitive neural reactions towards the cues (i.e. higher activity in the ventral striatum) [21]. In other words, despite explicit negative cognitions towards BSD because of the long-term negative consequences (see above), the pathological consumer behavior may be maintained by an attentional bias and implicit positive cognitions towards buying/shopping stimuli.

Research using Dot-probe tasks has found an attentional bias towards specific addiction-related cues in individuals with substance use disorders [39,40], gambling disorder [33], and Internet-gaming disorder [34]. Implicit associations towards addiction-related cues have been frequently measured with the Implicit Association Test [41], e.g. in individuals with gambling disorder [35], Internet-gaming disorder [36], Internet-pornography-use disorder [37] and in children and adolescents with Internet-use disorder [42].

Besides the cognitive processes described above, a person’s ability to withhold or stop a behavior is crucial in the development and maintenance of addictions [43]. Inhibitory control abilities have often been measured using Go/no-go tasks in which participants have to react or inhibit responses to addiction-related vs. neutral cues [44]. Deficits in inhibitory control have been demonstrated in patients with substance use disorders [45–47] and patients with gambling disorder [48]. Nicolai et al. [29] investigated inhibitory control abilities in relation to BSD in a convenience sample. They found that those individuals who exhibited more symptoms of BSD showed impaired performance in the Go/no-go task. The association between symptom severity of BSD and impaired inhibitory control was stronger in negative mood states [29].

In view of the proposed interplay of cognitive processes, the dual-process models framework in human decision-making has been related to BSD [38]. Dual-process models consider two neural systems: a fast, impulsive, intuitive system (subcortically located, rather automatic; reacting to immediate reward and punishment) and a slower, reflective system that consciously works through different considerations (prefrontally located, rather controlled; linked to conscious deliberations) [49]. Addictive behaviors may occur because the impulsive neural system is not down-regulated by the reflective neural system or overrides the reflective system due to drug-related neuroadaptations [50]. Referring to BSD, the confrontation with buying/shopping-related cues may predominantly stimulate the impulsive system (i.e. increase the decision for the short-term rewarding option of buying/shopping), while reflective processing is diminished (i.e. poor spending self-control) [38]. These assumptions are in line with other 2-factor models of BSD that refer to biologically driven conceptualizations of personality and temperament [51, 52]. According to past studies [53, 54], BSD is significantly related to 1) increased emotional reactivity (bottom-up regulation; i.e. increased behavioral inhibition/activation system reactivity) and 2) deficient effortful control (top down regulation; i.e. reduced self-control) (for review see [55]).

Taken together, patients with substance-related and addictive disorders are likely to show cue-induced craving, attentional bias, implicit positive cognitions and impaired inhibitory control related to cues associated with the respective addiction. Although knowledge about these processes will contribute to a more comprehensive understanding of the etiology of BSD and its overlap with, and differences to, substance-related and addictive disorders, relatively little effort has been devoted thus far to exploring these mechanisms. To address this research gap, the current study investigated cognitive processes and inhibitory control in a clinical sample of patients with BSD compared to a healthy control group. It was expected that patients with BSD would suffer from more severe symptoms of anxiety, depression and hoarding disorders than healthy control participants. In terms of cue-induced craving, attentional bias, implicit cognitive processes, and response inhibition, the following hypotheses were drawn based on the literature and the theoretical considerations above:

1. Patients with BSD will show more craving reactions towards buying/shopping-related cues and higher baseline craving for buying/shopping than healthy control participants.
2. In patients with BSD, the symptom severity of BSD will be related to craving reactions.
3. Patients with BSD will exhibit a higher attentional bias towards buying/shopping-related cues than healthy control participants within a Dotprobe task.
4. Patients with BSD will show more implicit associations to buying/shopping-related cues with positive emotions than healthy control participants within an Implicit Association Test.
5. Patients with BSD will show greater response inhibition deficits than healthy control participants in response to buying/shopping-related cues within a Go/no-go task.
6. Given the role of craving as a result of the conditioning process in addictions, the relationship between symptom severity of BSD and performance in the aforementioned experimental tasks will be moderated by craving reactions in patients with BSD.

## Materials and methods

### Participants and procedure

A priori power analysis using the software program G*Power, 3.1.9 [56] and assuming a medium effect size for group differences based on previous findings [47,57] indicated that a sample size of 35 individuals in each of the two groups (BSD-group, control group) is sufficient to reach an 80% power when employing the .05 criterion of statistical significance. Initially, 41 consecutive outpatients with BSD and 41 individuals without BSD (healthy controls, HC) were recruited. BSD was the primary mental health condition of all 41 outpatients and the reason for seeking psychotherapy treatment. While patients and control participants were exactly matched for gender, a deviation of ±1 year was allowed around age. Inclusion criteria for both groups were age ≥18 years and sufficient German language skills. Exclusion criteria for both groups were learning or developmental disorders, psychosis, mania, current substance use disorder (except tobacco), acute suicidal ideations, and sensory impairments. Meeting the Pathological Buying Screener threshold for BSD (total score >28; see below) [11] was an exclusion criteria for the control group and an inclusion criteria for the BSD-group. The diagnosis of BSD was confirmed via clinical interview in accordance with the operational diagnostic criteria for compulsive buying proposed by McElroy et al. [2]. The interviews were conducted by experienced psychologists/psychiatrist of the respective recruitment center (see below).

Patients were recruited at three different sites in Germany (Hannover Medical School *n* = 34, salus Clinic Friedrichsdorf *n* = 3, Center for Behavioral Addiction Research (CeBAR) at the University of Duisburg-Essen *n* = 2) and at the University Hospital Basel, Switzerland (*n* = 7). Control participants were recruited by word-of-mouth and notices in public venues (hospitals, cafés, supermarkets, libraries). Because this study focused on experimental tasks, participants with incomplete experimental data were dropped from the study, resulting in a final sample of 39 patients with BSD and 39 control participants.

**Fig 1:**
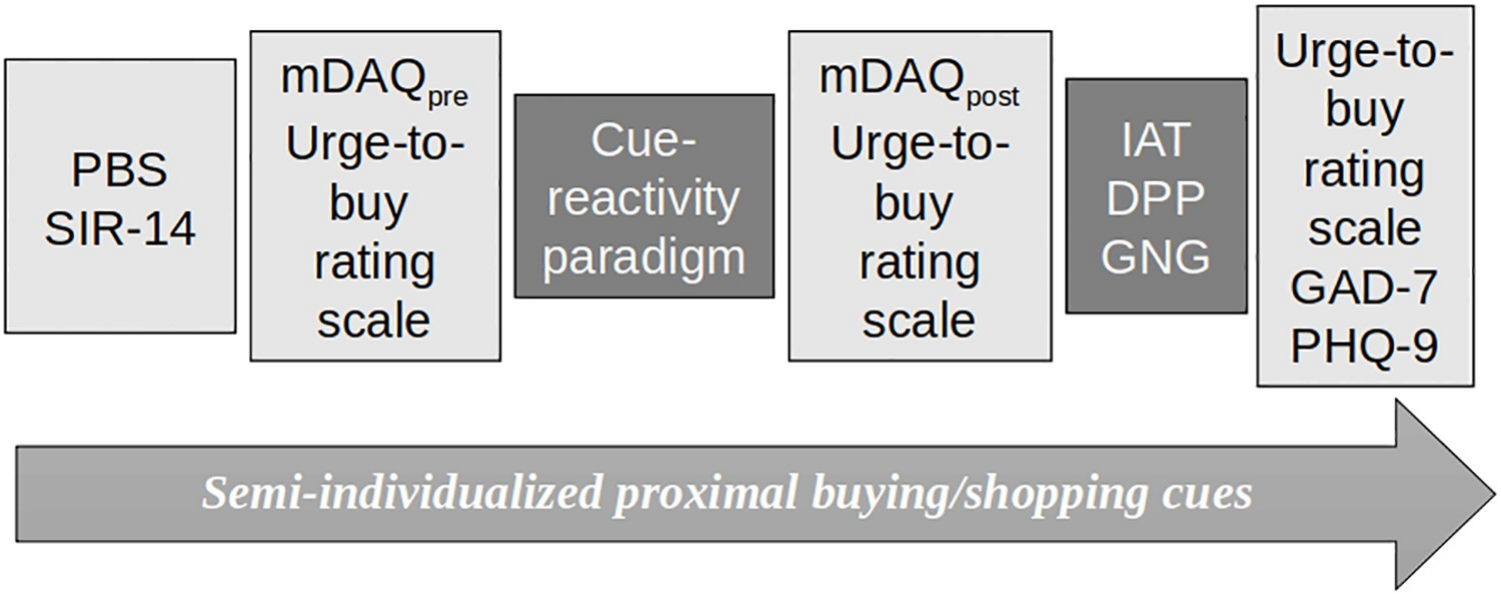
Study procedure. PBS = Pathological Buying Screener, SIR-14 = Saving-Inventory-Revised (without acquisition items), mDAQ = modified Desires of Alcohol questionnaire, GAD-7 = General Anxiety Disorder questionnaire, PHQ-9 = Patient Health Questionnaire depression scale.

Data were obtained between October 2016 and August 2017. Fig 1 illustrates the procedure. All participants provided information on sociodemographic variables. After answering several questionnaires (see below), they conducted a cue reactivity paradigm and performed three neuropsychological tasks. To avoid sequence effects, the tasks were administered in a freely randomized order. Measures for anxiety and depression were administered at the end of the testing. Data were collected electronically by using the open source software Lime Survey (Version 2.50, Lime Survey Inc., Hamburg, Germany) and were recorded on a local server.

The procedures were carried out in accordance with the Declaration of Helsinki. The Human Studies Committees of the Hannover Medical School approved this study (Ethical Approval No: 3360–2016), and the study protocol was subsequently approved by the department heads in each study location. Participation in the study was completely voluntary. Compensation of 25€ was provided for all participants enrolled in the study.

### Questionnaires

#### Buying-shopping disorder

Symptom severity of BSD was assessed by means of the Pathological Buying Screener (PBS) [11]. The questionnaire consists of 13 items that can be scored on a 5-point Likert scale from 1 (“never”) to 5 (“very frequently”). A cut-off score of >28 is considered indicative for BSD. The Cronbach’s α coefficient in the current study was .97.

#### Craving responses

Craving with respect to buying/shopping was measured using a modified version of the Desires of Alcohol questionnaire (mDAQ) [58] that has already been utilized in past studies on BSD [22,28]. The 14 items (e.g., ‘‘Going shopping would be pleasant now’, or ‘‘My desire to go shopping now seems overwhelming”) can be scored on a 7-point Likert scale from 0 (“complete disagreement”) to 6 (“complete agreement”). Higher mDAQ mean scores indicate higher subjective craving reactions. The questionnaire was administered before and after the cue-reactivity paradigm to assess baseline craving (mDAQpre; current study α = .98) and potential changes in craving within cue presentation (mDAQpost; current study α = .98). In addition, all participants were asked to rate their current urge to buy on a single item rating scale (from 0 = “no urge to buy” to 100 = “very strong urge to buy”). This question was presented before (t1) and after (t2) the cue-reactivity paradigm and after completing the experimental tasks (t3).

#### Psychiatric comorbidity

For means of comparison, assessment also included measures for anxiety, depressive and hoarding disorders. The 7-items of the General Anxiety Disorder (GAD-7) questionnaire enquire about symptoms of anxiety (current study α = .96) [59]. The 9-items of the Patient Health Questionnaire depression scale (PHQ-9) reflect each of the 9 DSM-IV criteria for depression (current study α = .92) [60]. The GAD-7 and the PHQ-9 items are answered on a 4-point Likert scale from 0 (“not at all”) to 3 (“nearly every day”). Symptoms of hoarding disorder were assessed with the German 19-item version of the Saving-Inventory-Revised (SIR) [61]. Items can be scored on a 5-point Likert scale from 1 (“strongly disagree”) to 5 (“strongly agree”). The questionnaire consists of the three subscales acquisition, difficulty discarding and clutter. For the current study, the acquisition subscale (5 items) was removed to avoid an overlap with BSD. Accordingly, only the subscales difficulty discarding (7 items) and clutter (7 items) were used to build the total score (SIR-14; current study α = .96).

### Experimental tasks

For all tasks, photographs of consumer goods (proximal cues) and buying/shopping scenes (distal cues) were used in accordance with previously performed buying/shopping-specific cue-reactivity paradigms [22,62].

#### Cue-reactivity paradigm

To semi-individualize the proximal visual cues with respect to the person’s buying/shopping preference, all participants were first asked to choose one out of eight buying/shopping categories (female categories: bags, books, CDs/DVDs, clothes, cosmetics, housewares, jewelry, shoes; male categories: books, CDs/DVDs, clothes, computer, electronic devices: hifi and tv, electronic devices: smartphone/photo, shoes, sporting articles). These 10 semi-individualized proximal visual cues together with 10 distal (not individualized) buying/shopping cues were then randomly presented in a size of 700 x 500 pixels via Presentation software (Version 20.0, Neurobehavioral Systems Inc., Berkley, CA, USA) and rated by the participants with regard to arousal (1 = “not at all arousing” to 0 = “very arousing”), valence (1 = “not pleasant” to 5 = “very pleasant”) and urge to buy (1 = “no urge” to 5 = “high urge”).

#### Dotprobe paradigm (DPP)

A visual DPP was used to measure participants’ attentional bias towards buying/shopping-related pictures. Participants were instructed to indicate the position of a dot (left- or right-sided) by pressing one of two response buttons on a standard keyboard as fast and accurately as possible. At the beginning of each trial, a central fixation cross was presented for 500 ms, followed by a 500 ms presentation of one buying/shopping-related and one neutral picture (respectively on the left and right side of the screen). After picture offset, a white dot probe (Arial, size 50, on a black background screen) was presented, either replacing the position of the buying/shopping-related or the neutral picture. The dot probe remained until the participant pressed one of the response keys. The BSD-related stimulus material consisted of the aforementioned 10 semi-individualized proximal visual cues and the 10 not-individualized distal buying/shopping cues. These cues were paired with 20 neutral object pictures (e. g. fire hydrants, ships) from the International Affective Picture System (IAPS) [63] taking into account the cues’ complexity. In addition, 40 IAPS pictures with a neutral valence were paired either with the 20 buying/shopping-related cues or the 20 neutral object IAPS pictures. Each picture pair was presented 4 times with counterbalanced side (left/right) and dotprobe location (left/right), resulting in 160 presentation trials. Only the pairings with buying/shopping-related pictures were analyzed. As dependent variable, the latency of response was recorded (in ms) and trials with response errors and reaction times <100 ms or >1000 ms were excluded. An attentional bias score was calculated for each participant by subtracting the mean latency (ms) to respond to a probe replacing a buying/shopping-related picture (congruent trial) from the mean latency to respond to a dot replacing a neutral picture (incongruent trials). Positive values for the attentional bias score suggest an orientation towards the buying/shopping-related visual cues. Presentation Software (Version 20.0, Neurobehavioral Systems Inc., Berkley, CA, USA) was used to present the stimuli and to record the behavioral responses.

#### Implicit Association Test (IAT)

A modified version of the IAT [41] was used to assess individual’s implicit (automatic) cognitions towards buying/shopping-related pictures. For the current study, a previously utilized version of the task was modified by using buying/shopping pictures (vs. jogging pictures) as targets. The prior study had focused on Internet-pornography-use disorder by using Internet-pornography vs. jogging pictures as targets [37]. The jogging pictures have been tested for neutrality by Snagowski et al. [37]. In the current study, participants were asked to categorize pictures as fast as possible into target concepts (“buying/shopping” vs. “jogging”) and attribute concepts (“positive” vs. “negative”) by using two buttons on a standard keyboard. In round 1 and 2, target (“buying/shopping pictures” vs. “jogging pictures”) and attribute concepts (“positive pictures” vs. “negative pictures”) were introduced and practiced. In round 3 and 4, target and attribute concepts were combined addiction congruently (“buying/shopping or positive” for one keyboard button vs. “jogging or negative” for the other button). In round 5, the response buttons for the target concept were exchanged and practiced again. In round 6 and 7, target and attribute concepts were combined addiction incongruently (“jogging or positive” vs. “buying/shopping or negative”). Within each concept category, ten pictures were presented in a randomized order. It is assumed that individuals with addictive disorders respond faster to congruent pairings (“buying/shopping or positive” vs. “jogging or negative”) than to incongruent pairings (“jogging or positive” vs. “buying/shopping or negative”). As dependent variable, the D_2SD_ score was used, since this algorithm was recommended by Greenwald et al. [64] and others [37,65]. The D_2SD_ value is computed as the difference in reaction times between the incongruent pairings and the congruent pairings divided by their overall standard deviation [64]. Higher D_2SD_ scores indicate stronger positive implicit associations with buying/shopping-related pictures than lower values. Presentation Software (Version 20.0, Neurobehavioral Systems Inc., Berkley, CA, USA) was used to present the stimuli and to record the behavioral responses.

#### Go/no-go task (GNG)

A modified version of the GNG shifting task [66] developed by Meule and Kübler [67] that has been previously used to test food-related response inhibition was administered. For the present study, buying/shopping-related or neutral landscape pictures were used as visual cues. The pictures were arranged into 16 blocks each including 20 trials in which either one of the semi-individualized proximal buying/shopping-related pictures (see cue-paradigm) or a neutral picture was presented (320 trials in total). Prior to each block, either buying/shopping-related or neutral pictures were defined as the target category. Participants were instructed to respond to pictures of the target category as fast as possible by pressing the space bar (go-trial), but to withhold their responses to pictures of the distractor category (no-go trial). Within each block, every picture was presented once for 500 ms in a randomized order. The inter-trial interval lasted 1000 ms and a blank screen (in case of right response) or a feedback screen (in case of false reaction or omission) was presented. Participants started either with buying/shopping-related or neutral pictures as target (counterbalanced across subjects). Every second block the target category changed, resulting in shift blocks in which participants had to change their stimulus-response association. A practice block of 20 trials was administered before starting the GNG. The complete GNG lasted about 15 min. Commission errors (CEs, i.e. failure to inhibit a response) either in the buying/shopping-related cues as target condition or the neutral cues as target condition were recorded. In order to combine both types of inhibition errors in one variable, the number of CEs in the neutral target condition was subtracted from the number of CEs in the buying/shopping-related cues as target condition. The resulting CE-bias score was used as dependent variable. Positive CE-bias scores indicate more inhibition errors in response to buying/shopping pictures as distractors in the neutral target condition, and negative scores reflect more inhibition errors to neutral pictures in the buying/shopping target condition. E-prime (Version 2.0, Psychology Software Tools Inc., Pittsburgh, PA, USA) was used to present the stimuli and to record the behavioral responses [66–68].

### Statistical analysis

Statistical analyses were conducted using IBM SPSS Statistics Version 24.0 (IBM Corp., Armonk, NY, USA). Group comparisons (BSD vs. HC) with respect to demographics and questionnaire data were conducted using independent *t*-tests and *X*^*2*^-tests, as appropriate. For each group, the relationships between variables (questionnaires and task performance) were examined by calculating two-tailed Pearson correlations.

With regard to the cue-reactivity paradigm, between-group comparisons of buying/shopping pictures ratings (i.e. arousal, valence, urge to buy) were controlled for baseline craving (mDAQ_pre_) as a covariate, using analysis of covariance. Within-group changes in craving were analyzed using dependent *t*-tests. Furthermore, a two-way within-subject analysis of variance (ANOVA) was performed with “time” as the within-factor (pre vs. post buying/shopping picture presentation in the cue-reactivity paradigm) and “group” as the between-factor (BSD, HC). The degrees of freedom were corrected when the assumption of variance homogeneity was violated according to Greenhouse-Geisser.

For the experimental paradigms (DPP, IAT, GNG), between-group comparisons were performed using independent *t*-tests. The analyses were subsequently controlled for potentially confounding variables such as education and psychiatric comorbidity (i.e. symptoms of hoarding, anxiety, depressive disorders) [69,70] using analyses of covariance (ANCOVA). To test potential cue category effects, additional analyses were performed for the DPP and GNG. Reaction times (DPP) / commission errors (GNG) were analyzed by using repeated measures ANOVAs with the between-subjects factor “group” (BSD, HC) and the within-subjects factor “trial condition” (congruent, incongruent) or “category” (buying/shopping related cues vs. neutral cues).

To determine if the order in which the DPP, IAT and GNG tasks were administered affected the results, separate ANCOVAs were performed for the DPP, IAT and GNG task. The dependent variables were the outcome variables of the respective paradigms. In all three ANCOVAs, “group” (BSD, HC) was entered as the fixed between-subject factor and the task order as random factor (three levels: task administered in the 1^st^, 2^nd^ or 3^rd^ order).

Interactions between task performance in the DPP, IAT or GNG and craving reactions as predictors of BSD severity were analyzed with hierarchical moderated regression analysis. Predictors were centralized prior to performing the regressions [71]. Furthermore, it was tested whether the relationship between symptoms of BSD (dependent variable: PBS scores) and task performance in the DPP, IAT or GNG was u-shaped by performing curve-linear regression analyses.

The significance level was set to *p* < .05; all tests were two tailed. Cohen’s *d* (*t*-tests), partial η^2^ (ANOVA, ANCOVA) and the Ф coefficient (*X*^*2*^-test) were used as effect size estimates [72].

## Results

### Demographics and psychopathology

Table 1 displays the group comparisons (BSD vs. HC) with regard to sociodemographic variables, symptoms of BSD, and psychiatric comorbidity. Each group had a median age of 48.00 years and consisted of 29 women (74.4%) and 10 men (25.6%). In accordance with the inclusion criteria, the BSD-group acknowledged more symptoms of BSD as measured with the PBS [51] than the HC-group. The latter group had completed more school years than the BSD-group. No between-group difference was found in terms of partnership status. The BSD-group admitted more symptoms of hoarding, anxiety and depressive disorders. The magnitude of the between-group differences in comorbid mental disorders was large (all Cohen’s *d* ≥ 1). Therefore, subsequent group comparisons were adjusted for these variables.

**Table 1:** Demographics and psychopathology.

	BSD-group	Control group	Test statistic		Effect size
	*n* = 39	*n* = 39		*p*	
Age years, *mean (SD)*	44.97 (10.83)	44.77 (10.59)	*t*_(76)_ = .08	.933	*d* = .02
Partnership status single, *n* (%)	20 (51.3)	13 (33.3)	*X*^*2*^_(1)_ = 2.57	.109	Ф = .18
School years, *mean (SD)*	11.36 (2.13)	12.54 (1.65)	*t*_(76)_ = 2.73	.008	*d* = .62
PBS	48.85 (9.83)	19.49 (3.16)	*t*_(76)_ = 17.76	< .001	*d* = 4.02
SIR-14	36.41 (14.97)	20.64 (4.49)	*t*_(76)_ = 6.30	< .001	*d* = 1.43
GAD-7	10.79 (4.46)	3.74 (2.75)	*t*_(76)_ = 8.40	< .001	*d* = 1.90
PHQ-9	12.03 (6.47)	3.95 (2.95)	*t*_(76)_ = 7.09	< .001	*d* = 1.61

BSD = buying-shopping disorder; PBS = Pathological Buying Screener, SIR-14 = Saving-Inventory-Revised (without acquisition items), GAD-7 = General Anxiety Disorder questionnaire, PHQ-9 = Patient Health Questionnaire depression scale

Information regarding the duration and negative consequences of BSD was available from 32 patients. They reported a median duration of the disease of 10 years (*mean* = 15.06, *SD* = 11.23, *range* 1–44 years). Four patients (12.5%) exhibited bankruptcy due to their BSD, 10 patients (31.2%) admitted delinquent behavior related to BSD and four patients (12.5%) underwent penal proceedings for BSD-related criminal behaviors.

### Subjective buying/shopping pictures ratings and craving responses

**Fig 2:**
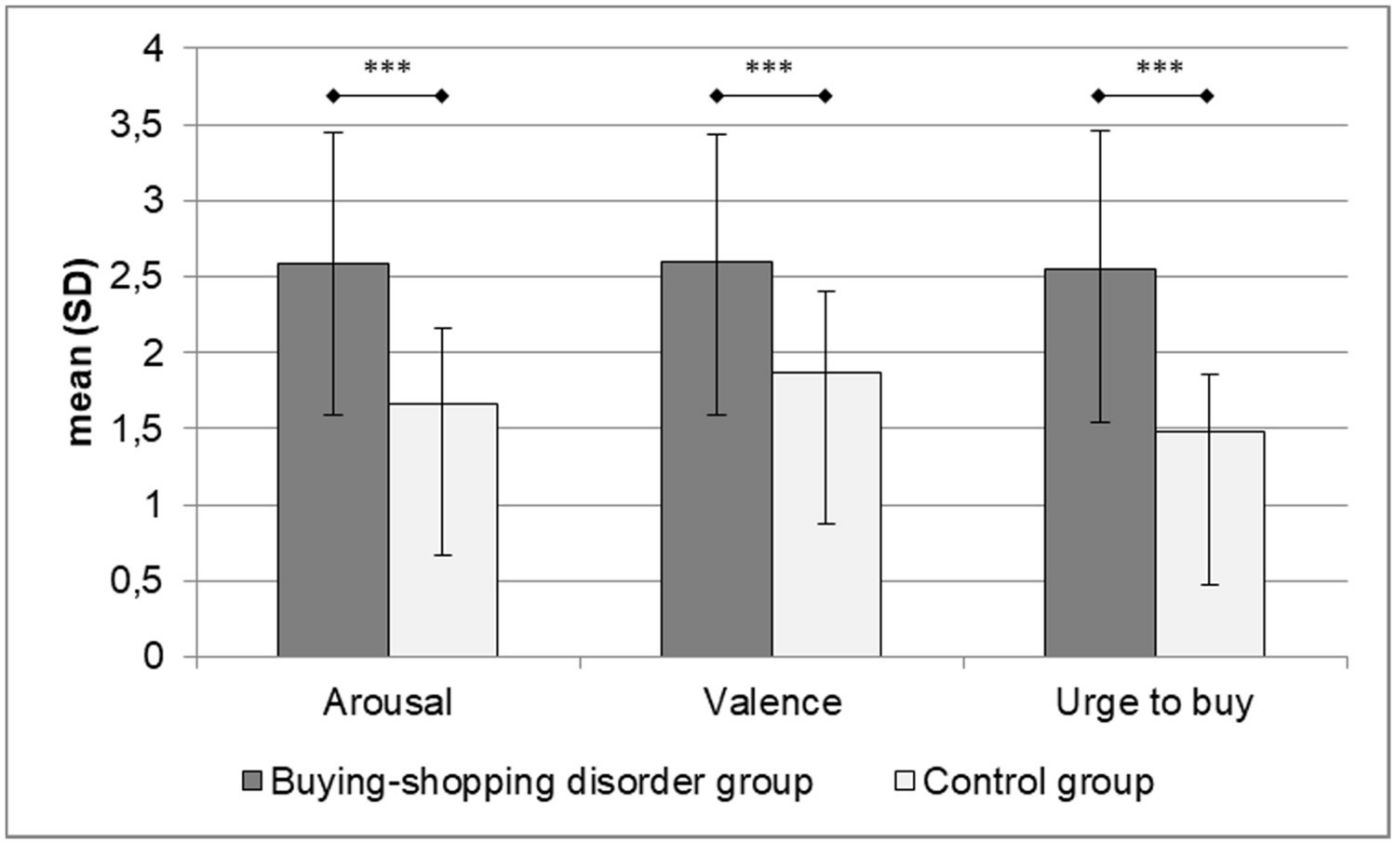
Subjective ratings of buying-shopping pictures. *** *p* < .001.

Results for the subjective ratings of buying/shopping pictures (i.e. arousal, valence, urge to buy) are shown in Fig 2. Compared to control participants, the BSD-group reported higher mean arousal (*t*_(76)_ = 5.75, *p* < .001, *d* = 1.30), more positive valence (*t*_(76)_ = 4.56, *p* < .001, *d* = 1.03) and a higher urge to buy related to the visual buying/shopping-related cues (*t*_(76)_ = 6.76, *p* < .001, *d* = 1.53). The group differences in subjective picture ratings were no longer significant when controlling for subjective baseline craving as measured with the mDAQ_pre_ (arousal: *F*_(1,75)_ = .88, *p* = .352, η_p_^2^ = .01; valence: *F*_(1,75)_ = .03, *p* = .863, η_p_^2^ = .00; urge to buy: *F*_(1,75)_ = 3.25, *p* = .075, η_p_^2^ = .04).

**Fig 3:**
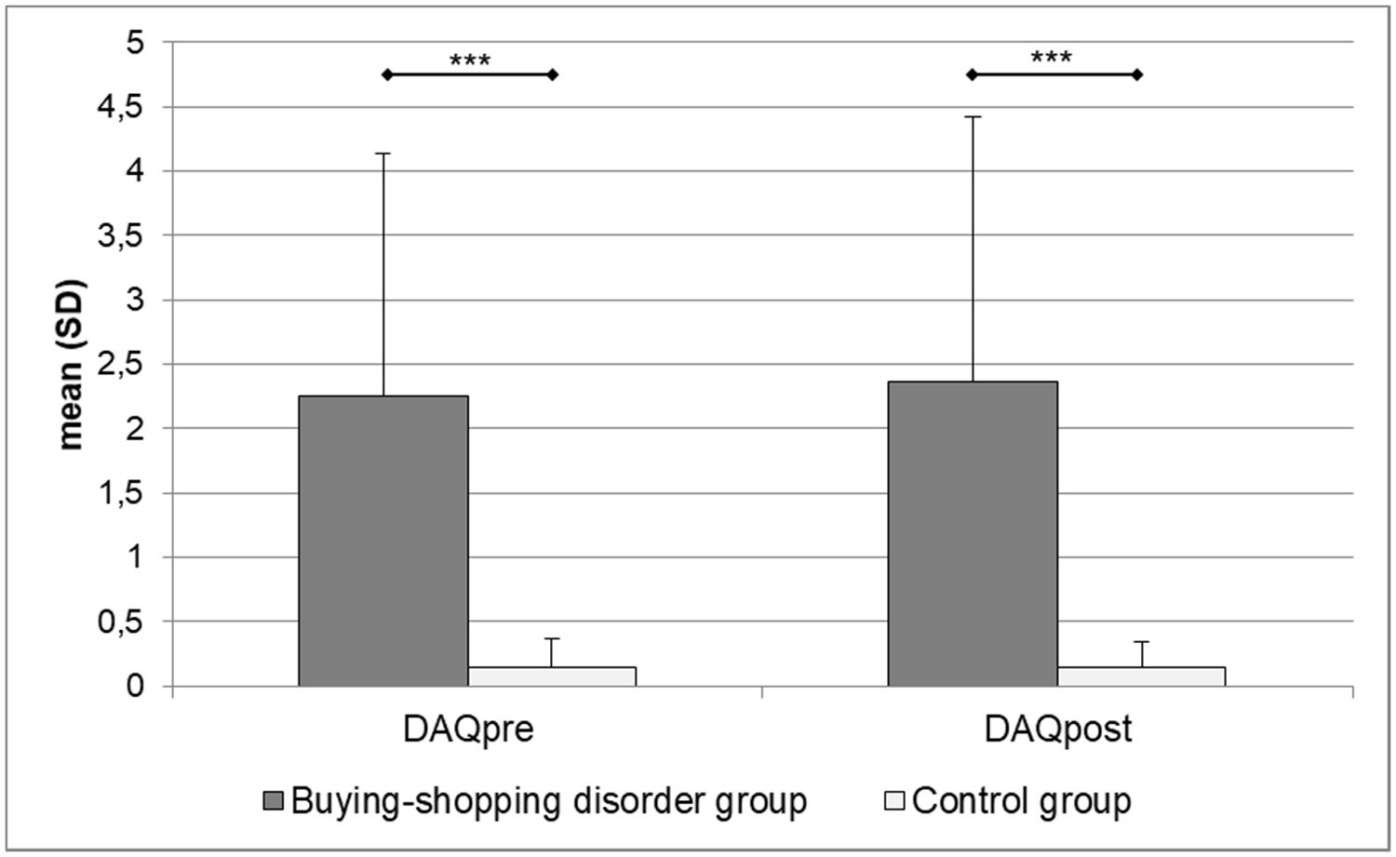
Subjective craving reactions pre and post buying/shopping pictures presentation. *** *p* < .001.

Fig 3 depicts subjective craving responses as measured with the mDAQ for the BSD- and the HC- groups. Patients with BSD reported higher subjective craving than control participants prior (*t*_(76)_ = 6.90, *p* < .001, *d* = 1.56) and following (*t*_(76)_ = 6.74, *p* < .001, *d* = 1.53) the buying/shopping pictures presentation. In both groups, craving did not change significantly across time (BSD: *t*_(38)_ = 1.47, *p* = .151, *d* = .26; CG: *t*_(38)_ = .17, *p* = .869, *d* = .03). A two-way within-subject ANOVA revealed a significant main effect of “group” (BSD, HC) (*F*_(1,76)_ = 47.25, *p* < .001, η_p_^2^ = .38) but no significant main effect of “time” (pre vs. post buying/shopping picture presentation) (*F*_(1,76)_ = 1.89, *p* = .174, η_p_^2^ = .02) and no significant “group x time” interaction (*F*_(1,76)_ = 2.13, *p* = .174, η_p_^2^ = .03).

In the BSD-group, current urge to buy as rated with the single item scale increased from t1 (*mean* = 50.79, *SD* = 32.93) to t2 (*mean* = 55.41, *SD* = 33.66) and decreased to t3 (*mean* = 40.31, *SD* = 34.02). Control participants rated their urge to buy as follows: t1 *mean* = 6.26 (*SD* = 9.76), t2 *mean* = 6.23 (*SD* = 9.46) and t3 *mean* = 4.08 (*SD* = 7.12).

### Relationship of BSD symptoms with cue reactivity variables, performance in experimental paradigms and comorbid psychopathology

Results of the bivariate Pearson correlations are listed in Table 2. As shown in the first section of Table 2, in patients with BSD, craving reactions were highly correlated with the symptom severity of BSD and moderately correlated with symptoms of comorbid mental health disorders. Symptoms of BSD were further positively—however weakly—correlated with attentional bias (DPP) but not with implicit associations (IAT) or decreased inhibitory control (GNG) related to buying/shopping pictures. In the HC-group, almost no significant correlations could be found (see Table 2). In both groups, experimental task performance was not related to psychiatric comorbidity.

**Table 2:** Two-tailed Pearson correlations (*r*) of buying-shopping disorder symptoms with craving variables, performance in experimental paradigms, and comorbid psychopathology.

	PBS	Arousal	Valence	Urge to buy	mDAQ_pre_	mDAQ_post_	DPP	IAT	GNG
**Buying-shopping disorder group (*n* = 39)**									
*Craving*									
**Arousal**	.78***								
**Valence**	.73***	.97***							
**Urge to buy**	.69***	.90***	.90***						
**mDAQ**_**pre**_	.73***	.79***	.74***	.83***					
**mDAQ**_**post**_	.69***	.80***	.75***	.82***	.97***				
*Experimental paradigms*									
**DPP**	.37*	.34*	.33*	.29	.28	.29			
**IAT**	-.01	-.12	-.08	-.12	-.08	-.08	-.18		
**GNG**	.27	.22	.16	.25	.31	.29	-.02	-.17	
*Comorbid psychopathology*									
**SIR-14**	.41*	.41**	.37*	.39*	.47**	.48**	.11	-.14	.24
**GAD-7**	.32*	.38*	.37*	.37*	.53**	.49**	< .01	-.14	.03
**PHQ-9**	.39*	.51**	.50**	.53**	.67***	.66***	.08	.12	-.09
**Control group (*n* = 39)**									
*Craving*									
**Arousal**	.21								
**Valence**	.22	.74***							
**Urge to buy**	.03	.71***	.64***						
**mDAQ**_**pre**_	.31	.50**	.21	.12					
**mDAQ**_**post**_	.25	.47**	.14	.23	.78***				
*Experimental paradigms*									
**DPP**	.08	.24	.08	.17	.07	.13			
**IAT**	-.05	.15	.03	.02	-.06	-.19	.09		
**GNG**	.13	.13	-.04	.25	.03	.01	-.15	.21	
*Comorbid psychopathology*									
**SIR-14**	-.04	.23	.30	.36*	-.22	-.15	-.09	.04	.15
**GAD-7**	.13	-.02	-.06	-.11	.11	.07	-.13	.04	.17
**PHQ-9**	.09	-.13	-.06	-.10	-.03	-.09	-.03	.04	.03

PBS = Pathological Buying Screener, mDAQ = modified Desires of Alcohol questionnaire, DPP = Dotprobe paradigm (attentional bias score), IAT = Implicit Association Test (D_2SD_), GNG = Go/no-go paradigm (commission error bias score), SIR-14 = Saving-Inventory-Revised (without acquisition items), GAD-7 = General Anxiety Disorder questionnaire, PHQ-9 = Patient Health Questionnaire depression scale.

* *p* < .05,

** *p* < .01,

*** *p* < .001.

### Group comparison of experimental task performance

Table 3 displays descriptive statistics of task performance in the DPP, IAT and GNG task separately for each group. No significant between-group differences were found with respect to the DPP attentional bias, IAT D_2SD_ and GNG commission error bias scores (see Table 3).

**Table 3:** Experimental task performance of patients with buying-shopping disorder (BSD) compared to control participants.

	BSD-group *n* = 39	Control group *n* = 39	Test statistic		Effect size
	*mean (SD)*	*mean (SD)*	*t*_(76)_	*p*	*d*
*Dotprobe paradigm*					
Attentional bias score	4.76 (19.56)	.91 (21.90)	.82	.416	.18
Reaction time in congruent trials^a^ [ms]	553.55 (124.15)	506.89 (91.18)	1.89	.062	.43
Reaction time in incongruent trials^b^ [ms]	558.31 (119.11)	507.80 (90.70)	2.11	.038	.48
*Implicit Associations Task*					
D_2SD_, *mean (SD)*	.24 (.48)	.06 (.49)	1.59	.116	.37
*Go/no-go task*					
Commission error bias, *mean (SD)*	1.44 (4.68)	0.97 (5.60)	.39	.694	.09
Total commission errors in response to buying/shopping-related cues	6.21 (3.95)	6.74 (4.43)	.57	.573	.12
Total commission errors in response to neutral cues	7.64 (6.37)	7.72 (4.50)	.06	.951	.01

^a^ dotprobe following buying/shopping-specific pictures,

^b^ dotprobe following neutral pictures.

With regard to the DPP, subsequent analyses showed no main effect of the repeated measures factor “trial condition” (congruent, incongruent) [Wilk’s λ *F*(1,76) = 1.46, *p* = .231, η^2^ = .02] and no interactions for “trial condition x group” [Wilk’s λ *F*(1,76) = .67, *p* = .416, η^2^ < .01]. The mean reaction time in incongruent trials was higher in the BSD-group than in the HC-group (*p* = .038, *d* = .48). There was also a trend towards slower reactions in congruent trials in patients with BSD compared to control participants that did not reach significance (*p* = .062, *d* = .43). Referring to the GNG, the positive CE-bias scores indicate more inhibition errors in response to buying/shopping-related pictures as distractors in the neutral target condition in both groups. There was a significant main effect of the repeated measures factor “category” (buying/shopping, neutral) [Wilk’s λ *F*(1,76) = 4.25, *p* = .043, η^2^ = .05) but no significant “category x group” interaction [Wilk’s λ *F*(1,76) = .16, *p* = .694, η^2^ < .01].

Separate ANCOVAs for each experimental task with “group” (BSD, HC) as between-subjects factor and “task order” as random factor were performed in the total sample. The results did not show significant task order administration or group effects on task performance. Also, the interaction “task order x group” was not significant (DPP: *F*_(2,72)_ = .97, *p* = .384, η_p_^2^ = .03; IAT: *F*_(2,72)_ = .18, *p* = .836, η_p_^2^ < .01; GNG: *F*_(2,72)_ = .31, *p* = .731, η_p_^2^ < .01). These findings were supported by the comparisons of smaller subsamples that were built depending on the order of task administration (i. e. groups that had performed the same task in 1^st^, 2^nd^ or 3^rd^ order). Task performances did not differ between patients with BSD and control participants across the subsamples (results not reported).

### Impact of craving on the relationship between symptom severity of BSD and performance in the DPP, IAT and GNG

Hierarchical moderated regression analyses were computed [71] to test the hypothesis that in patients the relationship between BSD severity (dependent variable: PBS score) and performance in the DPP, IAT or GNG is influenced by high craving (mDAQ_post_) or high subjective arousal/valence/urge to buy in response to BSD pictures. With respect to the HC-group, none of the applied regression models reached significance (results not reported). As can be seen in Table 4, in patients with BSD, craving variables were significantly associated with the PBS score, which resembles the results of the bivariate correlations (see above). However, no significant interaction effects could be found of craving or cue-reactivity with task performance in the DPP, IAT or GNG.

**Table 4:** Summary of regression analyses (step 3) investigating the impact of craving on the relationship between performance in experimental paradigms and severity of buying-shopping disorder (dependent variable: Pathological Buying Screener) in patients with buying-shopping disorder (*n* = 39).

Predictors	β	*t*	*p*	*R*^*2*^		β	*t*	*p*	*R*^*2*^		β	*t*	*p*	*R*^*2*^
DPP	.11	.74	.461		IAT	.13	1.14	.261		GNG	-.01	-.06	.953	
Arousal	.74	6.23	< .001		Arousal	.79	7.46	< .001		Arousal	.78	7.17	< .001	
DPP x Arousal	.00	.00	.999	.61	IAT x Arousal	-.11	-.98	.334	.62	GNG x Arousal	.13	.66	.511	.63
DPP	.097	.62	.542		IAT	.08	.70	.485		GNG	.01	.08	.940	
Valence	.655	5.11	< .001		Valence	.72	6.15	< .001		Valence	.73	6.10	< .001	
DPP x Valence	.072	.45	.658	.53	IAT x Valence	-.15	-1.29	.205	.54	GNG x Valence	.17	.85	.400	.56
DPP	.106	.68	.501		IAT	.14	1.07	.293		GNG	.08	.31	.755	
Urge to buy	.599	4.62	< .001		Urge to buy	.67	5.42	< .001		Urge to buy	.67	5.25	< .001	
DPP x Urge to buy	.140	.87	.388	.50	IAT x Urge to buy	-.17	-1.24	.223	.49	GNG x Urge to buy	.03	.14	.885	.49
DPP	.22	1.54	.133		IAT	.11	.86	.395		GNG	.19	.80	.428	
mDAQ_post_		4.91	< .001		mDAQ_post_	.68	5.63	< .001		mDAQ_post_	.67	5.31	< .001	
DPP x mDAQ_post_	-.09	-.62	.541	.50	IAT x mDAQ_post_	-.21	-1.65	.108	.50	GNG x mDAQ_post_	-.13	-.56	.581	.49

Arousal, Valence, Urge to buy = subjective buying/shopping pictures ratings, mDAQ = modified Desires of Alcohol questionnaire, DPP = Dotprobe paradigm (attentional bias score), IAT = Implicit Association Test (D_2SD_), GNG = Go/no-go paradigm (commission error bias score).

### Exploratory analyses of the relationship between symptom severity of BSD and performance in the DPP, IAT and GNG

To test whether the relationship between symptom severity of BSD and task performance in the experimental paradigms was not linear but u-shaped, additional curve-linear regression analyses were conducted with the PBS as dependent variable for the BSD- and the HC- groups separately. In the first step, the outcome variable of the respective task (DPP, IAT or GNG) was entered as independent variable. In the second step, the squared outcome variable of the respective task was entered. In the HC-group, neither significant linear nor significant u-shaped relationships between symptom severity of BSD and outcome variables of the DPP, IAT or GNG were found (results not reported). Below, the results concerning the BSD group are summarized.

With regard to the DPP, the attentional bias score was entered in the first step which significantly explained 13% of the variance of the PBS [*F* (1, 37) = 5.73, *p* = .022]. This result is in line with the Pearson correlation reported above (Table 2). In the second step, the squared attentional bias was entered; however, this did not explain variance of the PBS (only slight trend) [*R*^2^ = .15, Δ*R*^2^ = .02, *F* (2, 36) = 3.24, *p* = .051]. For the IAT, neither a linear [*R*^2^ < .01, *F* (1, 37) < .01, *p* = .971] nor a u-shaped relationship [*R*^2^ = .05, *F* (2, 36) = 0.72, *p* = .494] was found between the PBS and the D_2SD_. Similarly, there was no linear [*R*^2^ = .07, *F* (1, 37) = 2.91, *p* = .096] and no u-shaped [*R*^2^ = .11, *F* (2, 36) = 2.18, *p* = .128] relationship between the PBS and the CE-bias score of the GNG.

## Discussion

This case-control study investigated cognitive processes and inhibitory control ability in treatment-seeking patients with BSD compared to healthy control participants. The main findings are that patients with BSD reported more general craving for buying/shopping and stronger subjective craving reactions towards buying/shopping-related visual cues than the HC-group, but that they did not differ from control participants with regard to attentional bias, implicit cognitive associations and deficits of response inhibition toward buying/shopping-related cues. The outcome was not influenced by psychiatric comorbidity or task administration order.

The first part of the current results is in accordance with our first and second hypotheses, replicating the findings of Starcke et al. [62] and Trotzke et al. [22]. Patients with BSD responded with higher levels of subjective arousal, valence and urge to buy towards the buying/shopping-related visual cues than control participants. They further exhibited more general craving for buying/shopping than the HC-group before and after cue presentation and also after completing the experimental tasks (DPP, IAT, GNG). On a bivariate level, variables of cue reactivity and craving were highly correlated with the symptom severity of BSD in patients. Such correlations were not found in the HC-group. Given the importance of craving reactions in the development and maintenance of disorders due to substance use or addictive behaviors, these findings foster recent perspectives on the classification of BSD as a behavioral addiction [19,20]. As expected, the low-level craving for buying/shopping as measured with the mDAQ remained almost constant in the HC-group within the cue presentation (see Fig 2). It has to be noted that the slight increase in craving from pre to post cue presentation in the BSD group was also not significant. This finding might be explained by a possible ceiling effect given the elevated level of baseline craving in the patient group. As will be discussed later, alternative reasons for this finding could be the limited salience of some cues or a potential recruitment bias for the current study since the BSD group included patients chronically suffering from BSD (see below).

Contrary to the third, fourth and fifth hypotheses, no significant group differences were found with regard to the main outcome variables of the DPP, IAT and GNG. With regard to the DPP, it is noteworthy that patients with BSD responded more slowly in congruent trials (probe replacing a buying/shopping-related picture), and with a similar trend also in incongruent trials (probe replacing a neutral picture), than control participants. The high baseline craving for buying/shopping among patients might have impacted their attentional capacity leading to increased response latency.

The lack of differences in the main experimental tasks’ outcomes may reflect true absence of discrepant cognitive processes and a lack of difference in response inhibition in patients compared to control participants. That would mean that attentional bias, implicit positive cognitions and diminished inhibitory control are not connected to BSD, which is not consistent with our assumption regarding the overlap of BSD with substance-related disorders and other addictive behaviors. This preliminary conclusion requires careful discussion. Below, we consider alternative reasons for the lack of between-group differences in the experimental paradigms, e. g. sample characteristics, possible limitations with regard to the visual buying/shopping cues and aspects related to the phenomenology and course of BSD.

The diagnosis of BSD was confirmed by clinicians who are experienced in the field of addictive and impulse control disorders. Based on questionnaires, the patient group presented characteristics that are typical for BSD. In contrast to the control group, patients declared a high symptom severity of BSD and elevated levels of anxiety, depressive and hoarding disorder symptoms, which is consistent with our expectation and with the literature [1,3,15,16]. Given the self-reports regarding the duration and negative consequences of BSD (e. g. bankruptcy, delinquent behavior due to BSD) it seems plausible to assume that the current patient sample represented a clinical group with high symptom severity and chronicity of BSD. It should be considered that several patients may have undergone psychotherapy or counselling for BSD or have participated in self-help groups in the past. Those patients might have been trained to suppress urges to approach buying/shopping-related stimuli. In context of the dual-process model [38,50], training effects combined with the experience of negative consequences in everyday life due to BSD could promote an at least partial control of the reflective system over the impulsive system, which in turn could lead to buying/shopping-related avoidance tendencies. This assumption fits to the model proposed by Breiner et al. [73] for alcohol dependency, which indicates that not only approach but also avoidance tendencies are likely to occur in addicted patients. The former may be caused by positive expectancies, while the latter are probably promoted by negative expectancies [73]. The model of Breiner et al. [73] has been previously applied to explain the presence of both approach and avoidance tendencies towards pornographic stimuli in individuals with a propensity to Internet-pornography-use disorder [74]. With regard to the current study, it cannot be excluded that the lack of group differences in experimental tasks was caused by avoidance tendencies (instead of the expected approach tendencies) towards buying/shopping in the patient group. However, such reasoning remains speculative due to the lack of information about past treatments in the current sample.

In addition to a possible BSD-group bias, a possible HC-group bias should be taken into consideration. Some control participants may have perceived the buying/shopping pictures as more tempting than the neutral IAPS, jogging or landscape photos. In consumer societies, like Germany, many people take pleasure in shopping and spending. The omnipresence of advertising in everyday life may have contributed to attentional and cognitive biases (i. e. approach tendencies) towards buying/shopping-related cues in both patients and controls. The results of the GNG may support this assumption given that both patients and control participants showed inhibition problems when the targets were related to buying/shopping. However, the magnitude of the detected picture category effect in the GNG was weak (effect size η^2^ < .06). In addition, if the target pictures would have been tempting for the control participants, higher subjective arousal, valence and urge to buy ratings towards these pictures could have been expected in the HC-group.

According to reports of patients we have treated, BSD episodes are often inspired by current fashion advertising campaigns or by the desire to own the very latest product of a favorite brand. The suitableness of the current visual cues was established some years ago [22,62]. One may argue that some buying/shopping photos were limited with regard to currentness or attractiveness. However, patients with BSD exhibited more subjective craving reactions towards the buying/shopping pictures than control subjects, which implies that the visual cues were suitable (see Fig 2).

In addition to methodological aspects, the complexity of BSD should be taken into account when interpreting the results. Patients with BSD are a heterogeneous group with regard to clinical profiles, personality traits, psychiatric comorbidity, etc. [15,75–77]. In view of this potential heterogeneity and the aforementioned possible buying/shopping-related avoidance tendencies in patients with a longer history of BSD, analyses were conducted in order to test whether the relationship between symptom severity of BSD and performance in the experimental paradigms was rather curve-linear than linear. However, there was no u-shaped relationship between the variables under consideration.

Regardless of the heterogeneity, it is assumed that in the long run BSD serves as a strategy to cope with negative feelings, personal conflicts and stressful events [5,6,29,78]. According to patients’ reports, they almost always feel preoccupied with buying/shopping. Overwhelming urges to buy/shop that result in loss of control over spending are mostly related to external triggers (e. g. advertisements, commercials, etc.) and/or to the momentary feeling of discomfort due to perceived acute psychosocial stress (e. g. conflicts with their spouses, disappointments, humiliations, offenses, etc.). It has been shown that acute stress, through its influence on the prefrontal cortex, can promote the switch from a goal-directed to a habitual response to drug-related cues in individuals with substance use disorders [79]. Similarly to substance use disorders, it is possible that habitual positive cognitions towards buying/shopping and failures in self-control over the consumption of goods (i. e. approach tendencies; predominantly responding with the impulsive system that overrides the reflective system, see above) are most likely to occur in acute stress situations that result in negative mood [29]. Loeber et al. [80] have recently reported such an association with regard to a food-associated impairment of response inhibition in individuals with obesity and binge eating disorder. Unfortunately, the current study did not examine the potential influence of acute stress or momentary mood on cognitive processes and inhibitory control.

Taken together, the present study confirms previous research concerning the crucial role of craving—a feature of addictive behaviors—in BSD. Given that craving and cue-reactivity have been conceptualized as classically conditioned responses, cognitive-behavioral therapy (CBT) programs for BSD comprise sessions covering cue exposure techniques and response prevention [81–83]. However, more effort is needed to explore the specific effect of cue exposure in comparison to other interventions for BSD (e. g. psychoeducation, planned avoidance, emotion regulation, cognitive restructuring, financial counseling) [84]. Furthermore, virtual reality (VR) shopping environments may increase the impact of CBT interventions. It has been shown that VR environments are effective in decreasing craving for substance-related or gambling cues [85–87]. Studies investigating the feasibility and viability of a VR shopping paradigm for the use in repeated cue exposure to overcome BSD are warranted.

The assumption that attentional bias, implicit associations, and deficient inhibitory control are relevant in BSD could not be proven by the current findings. Future research should address the potential methodological shortcomings discussed above and investigate the proposed predictive role of acute psychosocial stress and momentary mood on cognitive processing and response inhibition in BSD.
